# Effects of acute high-Intensity resistance exercise on cognitive function and oxygenation in prefrontal cortex

**DOI:** 10.20463/jenb.2017.0012

**Published:** 2017-06-30

**Authors:** Hyukki Chang, Kyungae Kim, Yu-Jin Jung, Morimasa Kato

**Affiliations:** 1. Department of Human Movement Science, Seoul Women’s University, Seoul Republic of Korea; 2. Department of Health and Nutrition, Yonezawa Nutrition University of Yamagata Prefecture, Yonezawa Japan

**Keywords:** High-intensity resistance exercise, Stroop test, prefrontal cortex, cognition

## Abstract

**[Purpose]:**

Moderate-intensity exercise is known to be the best effective intensity to enhance cognitive function, including memory and learning. However, the effects of high-intensity exercise in comparison with moderate- intensity exercise on cognitive function remain controversial. The aim of this study was to investigate the effect of high-intensity resistance exercise on cognitive function.

**[Methods]:**

Thirty-six healthy female college students volunteered to participate in this study. The participants were divided into four groups: (i) control group (CON); (ii) high-intensity resistance exercise group (HIR); (iii) high-intensity aerobic exercise group (HIA); and (iv) combined moderate-intensity exercise group (MIC). Immediately prior to and after exercise, the solved number (SN) and reaction times (RT) in the Stroop test (neutral task, NT and incongruent task, IT), as well as the tissue oxygen index (TOI) in the left and right prefrontal cortex (PFC) were measured in all groups.

**[Results]:**

In the NT, both HIR and MIC groups showed significant improvements in SN and RT compared with the CON group. Meanwhile, performance in the HIA group was significantly attenuated compared with that in the MIC group. In the IT, only the MIC group showed a significant increase in SN and RT compared with the CON group. Furthermore, the TOI in the PFC (left PFC in the NT, and bilaterally in the IT) was significantly lower in the HIR group compared with that in the CON group.

**[Conclusion]:**

The results of this study show worse cognitive performance and decreased PFC oxygenation in high-intensity exercise compared with moderate-intensity exercise and controls. These results suggest that high-intensity exercise may not improve cognition as effectively as moderate-intensity exercise.

## INTRODUCTION

Physical exercise not only promotes cardiovascular, musculo-skeletal, and endocrine health, but also improves various functions of the brain (appetite and stress regulation, memory and cognitive function, and mood)^1, 2^. The effects and benefits of exercise vary depending on the intensity, duration, and type of exercise. Aerobic exercise performed at a moderate intensity is known to enhance cognitive function. More recently, studies have shown that low-intensity aerobic exercise may also help to improve cognitive function. In contrast, it has been reported that high-intensity aerobic exercise has the opposite effect on cognitive function, indicating that the cognitive benefits of aerobic exercise depend on exercise intensity^3^. However, the effects of resistance exercise on cognitive function remain to be determined. Furthermore, the effects of high-intensity aerobic and resistance exercise on cognitive function have rarely been compared. 

In animal studies, it has been reported that aerobic exercise leads to neurogenesis in the hippocampus, increased brain-derived neurotrophic factor (BDNF) production, and brain angiogenesis^4-6^. In contrast, human studies have been inconclusive, with some studies reporting improved cognitive function, and others showing a lack of significant improvement^7, 8^. These inconsistent results may be attributable to factors related to the participants in the study (psychological/ health status) or variations in exercise routines (intensity, frequency, place, etc.). Nevertheless, most studies have reported that aerobic exercise contributes to improved memory, attention, learning, cognitive speed and capacity, planning, problem solving, and decision-making. However, these benefits are dependent on exercise intensity, and it appears that high-intensity exercise beyond the optimal intensity level leads to an attenuation of the positive effects, showing an inverted U relationship^9^. 

Aerobic exercise of moderate intensity activates large muscles, which increases blood flow to the cerebrum and elevates hemoglobin levels, thus improving cognitive function^10^. The concentration of neurotransmitters in the brain also increases with exercise^9^. Nonetheless, positive changes in cerebral blood flow are dependent on exercise intensity. Furthermore, increased cerebral blood flow and neurotransmitter release during high-intensity exercise leads to an elevation in the level of stress beyond the anaerobic threshold, which subsequently interferes with cognitive function^9^. For this reason, most studies that have demonstrated the cognitive benefits of exercise have focused on moderate-intensity aerobic exercise. Some studies have shown marginal cognitive benefits of high-intensity aerobic exercise^7, 8^. For example, it was observed that reaction times in the Stroop test tended to increase in response to increasing exercise intensity, regardless of an individual’s fitness level^10^. However, it appears that high-intensity exercise temporarily affects executive control and increases performance errors. 

Resistance exercise is a type of physical exercise that uses external resistance to induce specific muscular contractions. In addition to its association with several health benefits, such as reduced risk for sarcopenia, metabolic diseases, and osteoporosis, and with improved quality of life, the large variety of strength-training equipment that has become widely available has contributed to the increasing popularity of resistance exercise^11^. High-intensity resistance training, which increases strength, is particularly beneficial for preventing musculoskeletal conditions in women with low bone density and muscular mass. In recent years, short-duration high-intensity exercise has become widely accepted among individuals aiming to include an exercise routine into their busy schedules. However, studies examining the cognitive effects of resistance exercise are scarce in comparison with those concerning aerobic exercise. Furthermore, most of the available study samples are limited to older individuals. Therefore, more research is needed to verify the cognitive benefits of high-intensity resistance exercise, which has become popular among younger adults. 

One previous study has examined the appropriate level of exercise intensity for older individuals who have an underlying condition such as Alzheimer’s or Huntington’s disease, while another has examined the effects of moderate exercise intensity on younger individuals^7, 11^. Nonetheless, studies examining the relationship between cognitive function and high-intensity resistance exercise (for muscular development) in young healthy participants are sparse. It is particularly important to examine the cognitive effects of increasingly popular high-resistance exercise in young women who need strength training. Even though high-resistance exercise is strongly recommended for young women, few studies have examined its effects on cognitive function^3^. 

In the present study, we used the Stroop test to assess changes in cognitive function. The Stroop test measures executive functions by determining reaction times to colors and letters, and involves prefrontal cortex (PFC) activation^12^. As performance on the Stroop test depends on PFC activation, the task performance can be used as an indirect measure of PFC activation^12^. The PFC is responsible for working memory, learning, emotional regulation, and selective information processing. Therefore, it is closely associated with cognitive function and is modulated by both acute aerobic exercise and resistance exercise^13-15^. The tissue oxygen index (TOI) is a biological measure of cognitive function reflecting hemoglobin levels (a measure of the extent of oxygenation in the PFC), and shows high sensitivity and specificity to intracerebral changes^16-18^. Brain activity leads to increased oxygen consumption, which is accompanied by an increase in cerebral blood flow due to neurovascular coupling^19^. 

The purpose of this study was to identify the effects of high-intensity resistance exercise compared with high-intensity aerobic exercise and combined moderate- intensity exercise on cognitive function measured with the Stroop test and TOI in the PFC of female college students. 

## METHODS

### Participants

Thirty-six healthy female college students in theirearly twenties were recruited to participate in this study(Table 1). In a preliminary survey, potential candidateswere selected using the following criteria: no history ofmajor physical or psychiatric illnesses, no use of medications, and normal vision and chromatic vision (for theStroop test). Prior to participation in the study, all participantsattended an information session, during whichthey were thoroughly briefed on the aim and proceduresof the study, and provided written consent. Upon submissionof consent, participants’ physical measurementswere obtained, their one repetition maximum (1RM)and heart rate reserve (HRR) were calculated, and threepractice sessions of the Stroop test were conducted priorto the actual test. 

**Table 1.** Participant demographic characteristics.

	age (year)	height (cm)	weight (kg)	BMI
CON (n=9)	21.8 ± 1.4	160.8 ± 4.1	52.2 ± 6.2	20.3 ± 3.1
HIR (n=9)	21.1 ± 1.6	162.1 ± 5.0	56.3 ± 6.3	21.4 ± 1.8
MIC (n=9)	20.4 ± 1.5	162.9 ± 5.5	56.4 ± 5.8	21.2 ± 1.3
HIA (n=9)	22.1 ± 1.4	166.0 ± 5.3	59.6 ± 5.7	21.6 ± 2.1

### Experimental design

To minimize the influence of physiological parameters on the outcome of the test, participants were instructed to avoid excessive physical activity and tobacco/alcohol/caffeine consumption in the 24 hours preceding the test. Testing and measurements were scheduled at least 4 hours after a meal, between 9AM and 6PM. The laboratory temperature was maintained at 23–25°C, with a humidity level below 60%. Noise levels were also controlled to ensure a quiet testing environment. 

**Figure 1.**
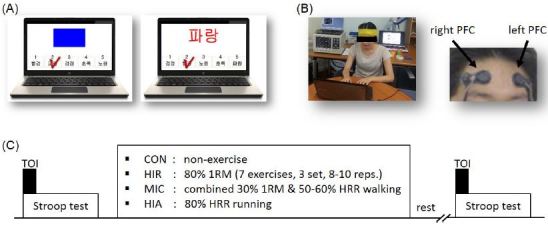
Experimental design: (A) Stroop test (neutral task and incongruent task); (B) tissue oxygen index (TOI) measurement in the prefrontal cortex (PFC) during the Stroop test; (C) control (CON), high-intensity resistance exercise (HIR; 7 exercises, 3 sets, 8–10 reps), combined moderate-intensity exercise (MIC; 7 exercises, 3 sets, 12 reps, and 30 min walking), high-intensity aerobic exercise (HIA; 30 min running). Resistance exercise comprised leg extension, lat pull down, leg curl, seated row, half squat, chest press, and arm curl.

Participants were randomly grouped into one of four groups: (i) controls (CON, n=9); (ii) high-intensity resistance exercise (HIR, n=9); (iii) moderate-intensity exercise combining resistance and walking (MIC, n=9); and (iv) high-intensity aerobic exercise (HIR, n=9). All participants assigned to an exercise group performed a warmup session for 10 min and conducted exercises specific to their group. Dependent variables were measured pre- and post-exercise. Post-exercise measurements were taken 15 minutes following the completion of exercise, the time after which heart rate returns to normal levels. The experimental design is shown in Fig 1. 

### High-intensity resistance exercise (HIR)

To establish the exercise intensity for this group, we employed the basic procedures suggested by Logan et. al.^20^. A week prior to the experiment, 1RM was measured while weight was increased, and high intensity was defined as 80% 1RM. The exercise routine for the HIR group consisted of three sets of seven exercises (leg extension, leg curl, lat pull down, seated row, squat, bench press, and arm curl), with 8–10 repetitions for each exercise and a work to rest ratio of 1:2. 

### Moderate-intensity exercise combining resistance and walking (MIC)

The MIC group routine consisted of low-intensity resistance exercise performed at 30% 1RM (12 repe-titions) and aerobic exercise (walking). The exercise was a combination of resistance- and aerobic-type exercises, conducted at approximately half of the exercise volume of the other groups (HIR and HIA). Participants in this group first completed resistance exercise and then performed 30 minutes of treadmill walking. The intensity of the walking exercise was maintained at 50–60% HRR, measured with a heart rate monitor (Polar, Finland). 

### High-intensity aerobic exercise (HIA)

The HIA group routine consisted of a 30-minute treadmill run performed at 80% HRR intensity, measured with a heart rate monitor. The treadmill speed was gradually increased. Participants in this group were thoroughly briefed on the potential risks of exercise prior to testing. In addition, their health and safety were continuously supervised throughout the exercise by monitoring the rating of perceived exertion at regular intervals. 

### Stroop test and TOI measurement

To measure cognitive function, we used a computer-based Stroop test program (designed by one of the researchers, H. Chang) in which a neutral task (NT) and an incongruent task (IT) were performed for 60 seconds each. We measured and compared reaction times (milliseconds) and the number of problems solved per 60 seconds. 

Measurements of TOI in the PFC were performed using near infrared spectroscopy (NIRS; NIRO-200, Hamamatsu, Japan). Sensors were connected to the left and right PFC, and changes occurring throughout the Stroop test were recorded. TOI was defined as the ratio of oxygenated hemoglobin to total tissue hemoglobin, and was expressed as follows: TOI = (HbO2/HbO2 + Hb)×100. The TOI was calculated from the slope of light attenuation along the distance from the emitting probe, and measured with the spatially resolved spectroscopy method. To reduce artifacts, participants were asked to minimize head and body movement. 

### Statistical analyses

The results of the Stroop test were represented as changes (pre-exercise score minus post-exercise score) for each group. To verify group differences, a one-way ANOVA was performed. When differences were observed, the Tukey test was performed as a post-hoc verification. To verify group differences in TOI during the Stroop test, an analysis of covariance was performed. All alpha levels for significance were set as p<0.05. Data processing was performed with SPSS 22.0 (Chicago, IL, USA). 

## RESULTS

### Stroop test (NT): changes in cognitive performance

**Figure 2.**
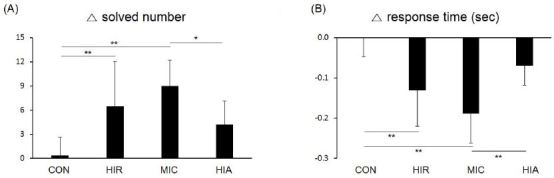
Changes in (A) solved number and (B) reaction time on the Stroop test (neutral task) during 60 seconds after protocol treatments in each group. Error bars are mean standard deviations. *p<0.05, **p<0.01

Fig. 2 shows the number of problems solved in 60 seconds by each group in the NT. The number of problems solved was larger in all exercise groups than in the CON group (0.33 ± 2.29) (Fig 2-A). However, a statistically significant increase was only observed in the HIR (6.44 ± 5.59, p<0.01) and MIC (9.00 ± 3.20, p<0.01) groups. The HIA group did not show a statistically significant increase compared with the CON group. In contrast, there was a significant difference in the number of problems solved between the MIC and HIA groups (4.22 ± 2.91, p<0.05). 

Reaction times improved in all groups compared with the CON group (-1 ± 47 ms, Fig. 2-B). However, the only statistically significant improvements were found in the HIR (-131 ± 90 ms, p<0.01) and MIC (-189 ± 74 ms, p<0.01) groups. The HIA group did not show a significant improvement compared with the CON group (-70 ± 49 ms). Reaction times in the MIC group were significantly higher than those in the HIA group (Fig. 2-B). 

### Stroop test (IT): changes in cognitive performance

**Figure 3.**
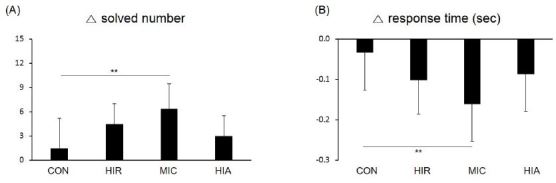
Changes in (A) solved number and (B) reaction time on the Stroop test (incongruent task) during 60 seconds after protocol treatments in each group. Error bars are mean standard deviations. **p<0.01

The number of problems solved in 60 seconds by each group before and after exercise is shown in Fig 3. All groups showed an improvement in the number of problems solved post-exercise compared with the CON group (1.44 ± 3.71). However, only the MIC group showed a statistically significant improvement (6.38 ± 3.07, p<0.01). 

Regarding reaction times, the HIR (-102 ± 84 ms) and HIA (-87 ± 93 ms) groups did not show a significant improvement compared with the CON group (-34 ± 93 ms), while the MIC group showed significantly shorter reaction times compared with controls (-162 ± 92 ms, p<0.01). 

### Altered TOI during the NT

**Figure 4.**
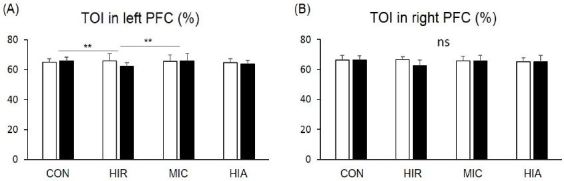
Tissue oxygen index (TOI) of (A) the left prefrontal cortex (PFC) and (B) the right PFC during the Stroop test (neutral task) in the 10 seconds after protocol treatments in each group. Error bars are mean standard errors. **p<0.01

We compared TOI during the 10 seconds following the start of the NT between the groups (Fig. 4), which showed significant group differences in the left PFC (p<0.05). The HIR group showed a significant decrease (65.9 ± 4.81 to 62.1 ± 2.59 %) compared with the CON (65.0 ± 2.37 to 65.8 ± 2.53 %, p<0.01) and MIC groups (65.5 ± 4.26 to 65.9 ± 4.70 %, p<0.01). The MIC and HIA groups (64.7 ± 2.57 to 63.8 ± 2.40 %) were not significantly different from the CON group. There was no significant intergroup difference for TOI in the right PFC (p=0.067). 

### Altered TOI during the IT

**Figure 5.**
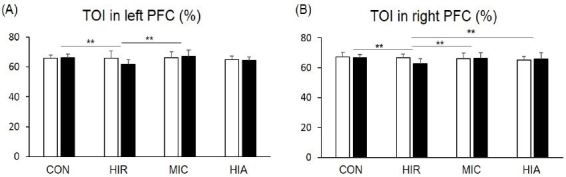
Tissue oxygen index (TOI) of (A) the left prefrontal cortex (PFC) and (B) the right PFC d uring the Stroop test (Incongruent task) in the10 seconds after protocol treatments in each group. Error bars are mean standard errors. **p<0.01

We compared TOI during the 10 seconds following the start of the IT between the groups (Fig. 5). Significant differences were observed between the groups in the left PFC. There was a significant decrease in TOI in the left PFC of the HIR group (65.7 ± 4.97 to 61.7 ± 3.10 %) compared with the CON (65.9 ± 2.11 to 66.2 ± 2.41 %, p<0.01) and MIC groups (66.1 ± 3.98 to 66.9 ± 4.28 %, p<0.01). There was no significant difference between the CON group and the MIC and HIA groups (65.0 ± 2.35 to 64.2 ± 2.53%). 

TOI was also significantly different between the groups in the right PFC (p<0.05). The HIR group showed a significant decrease (66.6 ± 2.61 to 62.7 ± 3.36 %) compared with the CON (67.4 ± 2.82 to 66.7 ± 2.29 %, p<0.05), MIC (66.1 ± 3.53 to 66.3 ± 3.69 %, p<0.01), and HIA groups (65.0 ± 2.46 to 65.7 ± 4.24 %, p<0.01). 

## DISCUSSION

Acute aerobic exercise facilitates cognitive function. However, the effects of resistance exercise on cognition remain unclear, although such exercise is an important component of exercise programs designed to improve health^21^. Generally, high-intensity resistance exercise is performed to stimulate muscular strength and muscle development, high-intensity aerobic exercise aims to potentiate cardiovascular health, and moderate-intensity combination exercise aims to improve overall health and fitness. Sequences consisting of aerobic exercises after resistance training and aerobic exercise on its own are commonly recommended for optimal health^22^. These two types of exercise complement each other and are suitable for individuals of all ages. Women who wish to start exercising regularly are typically advised to combine a low-intensity resistance routine and an aerobic routine. This type of exercise may be somewhat too low in intensity if the goal is to increase strength and cardiopulmonary capacity^12, 13^. However, because moderate- intensity combination exercise can be the most effective in improving cognitive function, it is suitable as a positive control and a reference point against which to measure the cognitive benefits of exercise. 

Cognitive function refers to brain functions underlying the storage and retrieval of information. As such, cognitive functions include memory, attention, judgment, execution, and calculation. Moderate-intensity aerobic exercise increases the brain size and improves cognitive functions^5, 9^. However, not all types of exercise offer the same level of cognitive benefit. In fact, the benefits vary according to the type and intensity of the exercise. Physical exercise is typically classified as aerobic exercise or resistance exercise. Moderate-intensity aerobic exercise is generally recommended to the public, as it offers a myriad of positive health effects, including improved cardiopulmonary capacity, fitness, reduced body fat, and enhanced cognition^3, 6, 9, 23^. 

In the present study, the effects of high-intensity resistance exercise on cognitive function were evaluated with the Stroop test and measures of TOI in the left and right PFC before and after a single bout of different types of exercise. Results from the Stroop test indicated that MIC was the most effective in improving both neutral and incongruent task scores. Moderate-intensity aerobic exercise helps distribute oxygen and nutrients throughout the brain through increased blood flow and oxygen saturation. In addition, increased oxy-Hb concentration in the PFC activates nerves and improves cognitive function^12, 24^. In contrast, the cognitive benefit of a single bout of resistance exercise was smaller than that of aerobic exercise^22^, and the cognitive benefit of high-intensity aerobic exercise was not different from that of control groups^23^. A lack of improvement or even reduction in cognitive function following high-intensity exercise has been shown in previous studies and is consistent with findings in the present study, in which the HIA group did not show a significant improvement in cognitive function. 

Given that cognitive function in the HIR group improved significantly compared with that in the CON group, cognitive benefits in this group were present but were the smallest observed in the study. Moreover, improvements in this group were significantly smaller compared with those in the MIC group. This may be because the measurements were taken immediately upon completion of a single bout of exercise, rather than the next day or following a chronic training regime. According to a previous study in which a group of healthy middle-aged subjects exercised at 80% 1RM for 24 weeks, the experimental group exhibited a far superior performance in cognitive function tasks involving long-term memory, executive control, and attention compared with the control group^25^. However, this result deviates from that of another study, in which a group of healthy elderly subjects subjected to a 12-week high-intensity resistance exercise regimen at 75–85% 1RM did not show an improvement in performance of tasks such as mental arithmetic^26^. In addition, a group of healthy college students who performed a single bout of high-intensity exercise at 80% 1RM did not exhibit significant improvements in cognitive function^27^. As seen in these studies, improved cognitive function appears to be highly dependent on the participants involved. In addition, because differences in exercise intensity and participant characteristics can yield slightly different outcomes, the fact that the present study involved female college students is of value. 

MIC, a combination of low-intensity resistance exercise and moderate-intensity aerobic exercise, showed a similar level of energy expenditure as HIR and HIA, and this exercise protocol was the most effective in improving cognitive function. The potential mechanism underlying the cognitive benefits of resistance exercise include the induction of increased IGF-1 concentration and blood flow to the brain^28^. A study by Mottola et al.^29^ using NIRS and hand grip maximal resistance exercise (30% Maximal Voluntary Contraction), reported a significant increase in oxy-Hb in the PFC. Pereira et al.^30^ have also reported that isometric knee extension exercises steadily increased PFC oxygenation until the lactic threshold was reached. Therefore, low- to moderate- intensity resistance exercise has a positive effect on indices associated with cognitive improvement. 

HIA was the least effective at improving cognitive functions compared with both MIC and HIR. Previous research suggests that the outcomes of high-intensity aerobic exercise can vary. For instance, a previous study reported that a single bout of aerobic exercise performed for 30 minutes at 60–70% VO2max improved cognitive function^22^. In contrast, another study examining the cognitive effects of a single bout of aerobic exercise on healthy subjects in their twenties reported that the error rate increased at 60–80% peak power, and that cognitive benefits of exercise were observed at low- to moderate-intensity^18^. Unlike other groups, participants in the HIA group performed the exercise until their heart rate reached 80% of VO2max, which is why activation of the sympathetic nervous system was more pronounced. Because activation of the sympathetic nervous system slows down executive function, the parasympathetic nervous system must be activated to compensate for this. For this reason, participants’ heart rates were measured 15 minutes following the completion of exercise. If the post-exercise measurements had been taken slightly later, the group’s cognitive improvements may have proved significant compared with the control group. Even so, high-intensity resistance exercise proved more effective at improving cognitive functions than did high-intensity aerobic exercise, at least immediately following the completion of exercise. For the IT, only the MIC group showed a significant improvement in cognitive functions. The IT may be slightly more challenging than the NT because it involves an additional selective improvement process (inhibition of task-irrelevant word and color recognition). The task likely engages several executive functions, including selective attention, inhibition, and cognitive flexibility^31^. In other words, when performing a more complex task, no significant cognitive improvement was observed in the HIR or HIA groups. These results in NT task suggest that HIR may have a particularly beneficial effect on cognitive function, because there was no effect on selective attention (IT task). 

We obtained slightly different results when comparing TOI between the groups. When performing the NT, neither the MIC nor the HIA group showed a significant difference compared with the CON group. A significant decrease in TOI was only observed in the HIR group. No significant group differences were observed for TOI in the right PFC. This is consistent with a previous study that showed increased activation of the left PFC following acute aerobic exercise, suggesting the existence of an exercise-related neural substrate in this region^12^. When performing the IT, significant group differences were observed in the bilateral PFC. Decrease TOI in the bilateral PFC was only observed in the HIR group compared with the CON group. The HIR group showed decreased TOI in the left PFC compared with that in the MIC group, and decreased TOI in the right PFC compared with that in the MIC and HIA groups. Therefore, only high-intensity resistance exercise acted to reduce TOI in both the left and right PFC. Thus, our findings suggest that long-term high-intensity resistance exercise may have negative effects on cognition. 

In conclusion, the results of the present study indicate that a single bout of high-intensity resistance exercise attenuates cognitive benefits associated with exercise, and this appears to be related to PFC oxygenation. A future study comparing the cognitive benefits obtained following long-term exercise versus single bouts of exercise could aid the design of an exercise program that supports cognitive improvement. 

## References

[1] Hötting K, Röder B. (2013). Beneficial effects of physical exercise on neuroplasticity and cognition. *Neurosci Biobehav Rev*.

[2] de Mello MT. (2015). Physical exercise, neuroplasticity, spatial learning and memory. *Cell Mol Life Sci*.

[3] Hillman CH, Erickson KI, Kramer AF. (2008). Be smart, exercise your heart: exercise effects on brain and cognition. *Nat Rev Neurosci*.

[4] Ando S, Kokubu M, Yamada Y, Kimura M. (2011). Does cerebral oxygenation affect cognitive function during exercise?. *Eur J Appl Physiol*.

[5] Colcombe SJ, Erickson KI. (2006). Aerobic exercise training increases brain volume in aging humans. *J Gerontol A Biol Sci Med Sci*.

[6] Soya H, Nakamura T, Deocaris CC, Kimpara A, Iimura M, Fujikawa T, Chang H, McEwen BS, Nishijima T. (2007). BDNF induction with mild exercise in the rat hippocampus. *Biochem Biophys Res Commun*.

[7] Audiffren M, Tomporowski PD, Zagrodnik J. (2009). Acute aerobic exercise and information processing: modulation of executive control in a Random Number Generation task. *Acta Psychologica*.

[8] McMorris T, Davranche K, Jones G, Hall B, Corbett J, Minter C. (2009). Acute incremental exercise, performance of a central executive task, and sympathoadrenal system and hypothalamic- pituitary-adrenal axis activity. *Int J Psychophysiol*.

[9] Kashihara K, Maruyama T, Murota M. (2009). Positive effects of acute and moderate physical exercise on cognitive function. *J Physiol Anthropol*.

[10] Labelle V, Bosquet L, Mekary S, Bherer L. (2013). Decline in executive control during acute bouts of exercise as a function of exercise intensity and fitness level. *Brain and Cognition*.

[11] Winett RA, Carpinelli RN. (2001). Potential health-related benefits of resistance training. *Prev Med*.

[12] Yanagisawa H, Dan I, Tsuzuki D, Kato M, Okamoto M. (2010). Acute moderate exercise elicits increased dorsolateral prefrontal activation and improves cognitive performance with Stroop test. *Neuroimage*.

[13] Chang Y-K, Etnier JL. (2009). Exploring the dose-response relationship between resistance exercise intensity and cognitive function. *J Sport Exerc Psychol*.

[14] Chang YK, Etnier JL. (2009). Effects of an acute bout of localized resistance exercise on cognitive performance in middle-aged adults: A randomized controlled trial study. *Psychol Sport Exerc*.

[15] Barella LA, Etnier JL, Chang YK. (2010). The immediate and delayed effects of an acute bout of exercise on cognitive performance of healthy older adults. *J Aging Phys Act*.

[16] Kubota Y, Toichi M, Shimizu M, Mason RA, Findling RL, Yamamoto K, Hayashi T, Calabrese JR. (2008). Altered prefrontal lobe oxygenation in bipolar disorder: a study by near-infrared spectroscopy. *Psychol Med*.

[17] Naulaers G, Meyns B, Miserez M, Leunens V, Van Huffel S, Casaer P, Weindling M, Devlieger H. (2007). Use of Tissue Oxygenation Index and Fractional Tissue Oxygen Extraction as Non-Invasive Parameters for Cerebral Oxygenation. *Neonatology*.

[18] Perrey S. (2012). NIRS for Measuring Cerebral Hemodynamic Responses During Exercise. *Functional neuroimaging in exercise and sport sciences*.

[19] Davranche K, Casini L, Arnal PJ, Rupp T, Perrey S. (2016). Cognitive functions and cerebral oxygenation changes during acute and prolonged hypoxic exposure. *Physiol Behav*.

[20] Logan P, Fornasiero D, Abernethy PJ, Lynch K. (2000). *Protocols for the assessment of isoinertial strength*.

[21] Williams MA, Haskell WL, Ades PA, Amsterdam EA. (2007). Resistance exercise in individuals with and without cardiovascular disease: 2007 update. *Circulation*.

[22] Ratamess NA, Kang J, Porfido TM. (2016). Acute Resistance Exercise Performance Is Negatively Impacted by Prior Aerobic Endurance Exercise. *J Strength Cond Res*.

[23] Erickson KI, Miller DL, Roecklein KA. (2012). The aging hippocampus: interactions between exercise, depression, and BDNF. Neuroscientist. *Neuroscientist*.

[24] Tomporowski PD. (2003). Effects of acute bouts of exercise on cognition. *Acta Psychologica*.

[25] Cassilhas RC, Viana V, Grassmann V. (2007). The impact of resistance exercise on the cognitive function of the elderly. *Med Sci Sports Exercise*.

[26] Tsutsumi T, Don BM, Zaichkowsky LD, Delizonna LL. (1998). Physical fitness and psychological benefits of strength training in community dwelling older adults. *Appl Human Sci*.

[27] Pontifex M, Hillman C, Fernhall BO. (2009). The effect of acute aerobic and resistance exercise on working memory. *Medicine & Science in Sports & Exercise*.

[28] Borst SE, Vincent KR, Lowenthal DT. (2002). Effects of Resistance Training on Insulin-Like Growth Factor and its Binding Proteins in Men and Women Aged 60 to 85. *J Am Geriatr Soc*.

[29] Mottola L, Crisostomi S, Ferrari M. (2006). Relationship between handgrip sustained submaximal exercise and prefrontal cortex oxygenation. *Adv Exp Med Biol*.

[30] Pereira M, Gomes P. (2009). Acute effects of sustained isometric knee extension on cerebral and muscle oxygenation responses. *Clin Physiol Funct Imaging*.

[31] Buck SM, Hillman CH, Castelli DM. (2008). The relation of aerobic fitness to stroop task performance in preadolescent children. Medicine & Science in Sports & Exercise.

